# Intelligence and language outcomes in school-aged children who are HIV-exposed, uninfected: the role of sex, perinatal risk factors, and socioeconomic status

**DOI:** 10.3389/fped.2025.1540420

**Published:** 2025-07-17

**Authors:** Leila Kahnami, Mary Lou Smith, Ari Bitnun, Jason Brophy, John G. Sled, Elka Miller, Jennifer Bowes, Mariève Hurtubise, Lena Serghides, Julia M. Young

**Affiliations:** ^1^ Department of Psychology, The Hospital for Sick Children, Toronto, ON, Canada; ^2^ Department of Psychology, York University, Toronto, ON, Canada; ^3^ Neurosciences and Mental Health Program, Research Institute, The Hospital for Sick Children, Toronto, ON, Canada; ^4^ Department of Psychology, University of Toronto Mississauga, Mississauga, ON, Canada; ^5^ Division of Infectious Diseases, The Hospital for Sick Children, Toronto, ON, Canada; ^6^ Department of Pediatrics, University of Toronto, Toronto, ON, Canada; ^7^ Division of Infectious Diseases, Children’s Hospital of Eastern Ontario, Ottawa, ON, Canada; ^8^ Department of Pediatrics, University of Ottawa, Ottawa, ON, Canada; ^9^ Translational Medicine Program, Research Institute, The Hospital for Sick Children, Toronto, ON, Canada; ^10^ Department of Medical Biophysics, University of Toronto, Toronto, ON, Canada; ^11^ Department of Diagnostic and Interventional Radiology, The Hospital for Sick Children, Toronto, ON, Canada; ^12^ Department of Medical Imaging, CHEO, University of Ottawa, Ottawa, ON, Canada; ^13^ Toronto General Hospital Research Institute, University Health Network, Toronto, ON, Canada; ^14^ Department of Immunology and Institute of Medical Sciences, Toronto, ON, Canada

**Keywords:** HIV-exposed uninfected, neurodevelopment, working memory, intellectual abilities, language abilities, developmental trajectory

## Abstract

**Background:**

Children who are HIV-exposed uninfected (CHEU) are at increased risk for neurodevelopmental impairments. Most studies report on neurodevelopmental outcomes in the first 2 years of life, with limited data available for school-aged CHEU. This interim study examined the intellectual and language outcomes in school-aged CHEU compared to children who are HIV-unexposed uninfected (CHUU).

**Setting:**

CHEU and CHUU aged 6–10 years recruited at two sites in Ontario, Canada.

**Methods:**

Intellectual and language abilities were measured using the WISC-V and CELF-5. Generalized linear models investigated associations of HEU-status with each neurodevelopmental outcome. An interaction term with sex was included to assess sex-specific effects. Gestational age, being small for gestational age (SGA), and household income were investigated as covariates.

**Results:**

65 CHEU (35 female, median age 9.00 years) and 42 CHUU (18 female, 8.96 years) were included. HEU-status was associated with significantly lower working memory and expressive language scores. In males, HEU-status was associated with lower scores on working memory, processing speed, overall intelligence, core, and expressive language abilities. No significant differences were observed in females by HEU-status. Household income was associated with all measures of intelligence and language. Lower working memory scores persisted in male CHEU after adjusting for covariates.

**Conclusion:**

Male CHEU and those with lower household income were the most vulnerable to cognitive and language deficits. Working memory deficits in CHEU indicates a specific cognitive vulnerability due to HEU exposure status. Our findings highlight the need for early interventions, including ensuring financial security and close neuropsychological follow-up.

## 1 Introduction

Globally, there are more than 16 million children who are HIV-exposed but uninfected (CHEU) (1). In Canada, between 200 and 250 CHEU are born each year (2). Most of these children are also perinatally exposed to HIV antiretroviral therapy (ART). *In utero* exposure to HIV (3), and HIV-related mechanisms such as maternal immune activation and inflammation (4, 5), as well as ART exposure (6), may place CHEU at a higher risk of perinatal complications and developmental deficits (7). Developmental vulnerabilities have been reported in CHEU, including differences in early neuropsychological development compared to children who are HIV-unexposed and uninfected (CHUU) (8). Perinatal risk factors are known to influence the trajectories of the developing brain (9, 10). However, there is a limited understanding of the impact of the perinatal risk factors on the neuropsychological development of CHEU in later childhood.

CHEU are at increased risk of being born small for gestational age, having low birth weight, being born preterm, experiencing neonatal jaundice, and being admitted to the neonatal intensive care unit (NICU) (7, 11–13). While preterm birth, low birth weight, and admission to NICU have been associated with an increased risk of neurodevelopmental delays and cognitive challenges in all children (9, 10, 14–16), reports of these associations in CHEU are limited to the ages of 6–24 months, and data in older CHEU are lacking (11, 17). In addition, CHEU are at increased risk of intellectual (18) and language challenges (19, 20). Despite these insights, the literature remains fragmented, with studies suggesting no significant differences in the neurodevelopmental trajectory and language development of CHEU up to the age of 5 (11, 17, 18, 21), while longitudinal studies suggest that differences in overall intellectual and expressive and receptive language abilities between CHEU and CHUU may become more prominent over time (8, 22, 23). Moreover, while sex differences in the neurodevelopmental trajectory of CHEU have been considered in younger age groups, little is known about the emergence of sex differences in older age groups (24, 25).

The existing literature includes a combination of low- and high-income countries, with limited published data from high-income countries, such as Canada. Research examining sex differences in the neurodevelopmental trajectory of CHEU remains limited. Further, previous studies have explored these associations in younger age groups. The cognitive outcomes of CHEU and the role of perinatal, demographic, and socioeconomic factors on intellectual and language abilities in school-aged CHEU remain unclear. As part of a Canadian multidisciplinary study, this current study examined the impact of HIV-exposed uninfected (HEU) status on the cognitive outcomes in school-aged children. Our aim was to compare the intellectual and language abilities of school-aged CHEU and CHUU, while considering the differences in demographic and socioeconomic characteristics and perinatal risk factors to determine potential moderating factors influencing these cognitive outcomes.

## 2 Methods

### 2.1 Study population

The findings of this study are an interim analysis of the Kids Imaging and Neurocognitive Development (KIND) study, an ongoing Canadian prospective cohort study of CHEU and CHUU designed, with target sample size of 180 CHEU and 65 CHUU, to assess the long-term neurodevelopmental health of these children, at two time points across a two-year interval. The KIND study enrolls children between the ages of 6–10 since 2020 across two clinical sites—The Hospital for Sick Children (SickKids) in Toronto and Children’s Hospital of Eastern Ontario (CHEO) in Ottawa. The key inclusion criteria for the CHEU group were being born to a mother living with HIV with a known exposure to ART, and having negative HIV status (defined as two or more negative DNA PCR assays performed at or after one month of age or negative HIV serology at any age). The CHUU were recruited from the community and school programs operating in areas of similar socioeconomic status as the families of CHEU, and through word of mouth from participants. Group matching criteria included age, ethnic background, household income, caregiver education level. The inclusion criteria for CHUU were having a mother with HIV-negative status during pregnancy. Exclusion criteria for both groups included a history of previous developmental or neurological conditions (e.g., stroke, Down’s syndrome) that were not related to HIV/ART exposure (in the CHEU group), exposure to maternal smoking (>1 cigarette/day), regular alcohol consumption (>3 drinks/week for >1 month), or any substance use during pregnancy.

### 2.2 Ethics statement

The Institutional Review Boards of The Hospital for Sick Children, Children’s Hospital of Eastern Ontario, and University Health Network approved the protocol. All participating families provided written informed consent, and children provided assent to participate in the study.

### 2.3 Demographic and perinatal measures

Demographic and perinatal health information was collected through structured parent interviews for both groups and a review of hospital medical records for CHEU. Demographic and perinatal information was self-reported by the mothers throughout the structured interview. Information regarding maternal HIV status and ART usage during the relevant pregnancy for the CHEU group was collected from medical records. The following demographic measures were obtained through the structured interviews: family income, maternal race, maternal education level, and languages spoken at home. A translator was used, where needed, with parents who did not primarily speak English.

Our primary exposure of interest was HEU-status (CHEU vs. CHUU). Perinatal measures of interest included: birthweight, birth weight centiles [determined using the INTERGROWTH-21st calculator (26)], small for gestational age (SGA) status (below the 10th birth weight centile), gestational age at birth, prematurity (born at <37 weeks gestational age), NICU admission, and birth complications (e.g., jaundice, infection, induced delivery).

### 2.4 Neurodevelopmental outcomes

The primary outcome measures were scores from two psychological assessment measures the Wechsler Intelligence Scale for Children—Fifth Edition [WISC-V (27)] and the Clinical Evaluation of Language Fundamentals- Fifth Edition [CELF-5 (28)]). These measures were administered by experienced research staff under the supervision of a psychologist or directly by psychologists. Indices of Verbal Comprehension, Visual-Spatial, Fluid Reasoning, Working Memory, Processing Speed, and Full-Scale IQ were obtained through the WISC-V. Indices of Expressive Language, Receptive Language, and Core Language abilities (combination of expressive and receptive language skills) were obtained from the CELF-5. Standardized scores were derived for each measure, with a population mean of 100 corresponding to the 50th percentile of typical development, and a standard deviation (SD) of 15.

### 2.5 Statistical analyses

Chi-square (*χ*^2^ tests) were used to compare demographic characteristics between CHEU and CHUU groups. The mean standard scores and 95% confidence intervals of the intelligence and language outcomes were calculated by HEU-status (CHEU vs. CHUU), and stratified by sex. The proportion of children scoring within the clinically impaired range (2 SD below the mean standardized score, i.e., <70) on each measure was calculated by HEU-status. Chi-squared tests were used to compare the proportions of clinically impaired scores between the two groups. Generalized linear models with robust standard errors (29) were used to examine the associations between each neurodevelopmental outcome and HEU-status. To investigate sex-specific effects models including an interaction term of HEU-status and sex were also used.

Exploratory Pearson’s correlation coefficients were first calculated to examine relationships between demographic, socioeconomic, and perinatal measures with each neurodevelopmental outcome. Perinatal factors included in the models were chosen *a priori*. Household income and maternal education levels were considered to represent socioeconomic status. To investigate how these perinatal factors and socioeconomic factors modified the associations between HEU-status with each neurodevelopmental outcome, subsequent generalized linear regression models included chosen perinatal factors and socioeconomic factors as independent variables. Gestational age at birth and birthweight were considered due to the significant differences in these factors between the CHEU and CHUU, but to avoid collinearity due to their high correlation (*r* = 0.70), gestational age at birth was selected. Birthweight centile, prematurity, NICU admission, and birth complications were excluded due to high correlations with gestational age at birth (Supplementary Table 1). Being SGA at birth was retained based on its association with neurodevelopmental outcome measures and low correlation with gestational age at birth (*r* = 0.14). Two models were evaluated to examine the impact of gestational age at birth and SGA. The first model adjusted only for gestational age at birth (Supplementary Table 2). The second model adjusted for gestational age at birth and being SGA (Supplementary Table 3) and was identified as having the best fit for perinatal factors. The models also included a “HEU-status by sex” interaction term. To account for socioeconomic status, household income was chosen due to its correlations with the neurodevelopmental outcome measures. Household income was included in the final model that included the perinatal factors of gestational age at birth and SGA. Model fit was compared using the Akaike Information Criterion (AIC) and Bayesian Information Criterion (BIC). All analyses were performed using R (v4.2.1) (30), STATA (v13) (31), and Prism (v9) (32), with statistical significance set at *p* < 0.05.

## 3 Results

### 3.1 Baseline demographic and perinatal measures

A total of 114 children were enrolled between January 2020 and April 2024. After excluding participants (*n* = 7) due to invalid results on the neurodevelopmental measures, 107 children (65 CHEU and 42 CHUU) were available for the analysis. Demographic and perinatal characteristics are shown in Table 1. Both groups had similar age (median age of 9.00 years for CHEU vs. 8.96 years for CHUU) and sex distribution (CHEU 53.8% girls vs. CHUU 42.9% girls). CHEU and CHUU were similar in maternal and socioeconomic characteristics. The majority of mothers identified as African/Caribbean/Black (70.3% of CHEU and 45.2% of CHUU). English was the primary language spoken at home for the majority of participants (89.1% for CHEU vs. 92.9% for CHUU). The majority of mothers had a college degree level education (42.9% of CHEU mothers vs. 47.6% of CHUU mothers). There were no statistically significant differences between groups in household income.

**Table 1:** Demographic and perinatal characteristics.

Characteristics	CHEU median (IQR) or *n* (%)	CHEU *n*	CHUU median (IQR) or *n* (%))	CHUU *n*	*p*-value
Child factors		65		42	
Age (years)	9.00 (7.25, 9.92)		8.96 (7.17, 10.08)		0.57
Sex					
Male	30 (46.2%)		24 (57.1%)		0.27
Female	35 (53.8%)		18 (42.9%)		
Maternal and SES factors					
Mother’s race		64		42	
Black African Caribbean	45 (70.3%)		19 (45.2%)		0.11
Caucasian	12 (18.8%)		14 (33.3%)		
Other	7 (10.3%)		9 (21.4%)		
Language spoken at home		64		42	
Primarily English	57 (89.1%)		39 (92.9%)		0.36
English and other	7 (10.9%)		3 (7.1%)		
Maternal Education		63		42	
Did not finish high school	6 (9.52%)		3 (7.1%)		
High school degree	14 (22.2%)		5 (11.9%)		0.68
College degree	27 (42.9%)		20 (47.6%)		
University undergraduate degree	8 (12.7%)		7 (16.7%)		
Post-university undergraduate degree	8 (12.7%)		7 (16.7%)		
Household Income		62		42	
Less than $25,000	17 (27.4%)		5 (11.9%)		0.092
$25,000–$49,999	21 (33.9%)		11 (26.2%)		
$50,000–$74,999	10 (16.1%)		13 (31.0%)		
$75,000–$99,999	5 (8.06%)		2 (4.76%)		
Over $100,000	9 (14.5%)		11 (26.2%)		
Perinatal Factors					
Birth Weight (kg)	2.90 (2.38, 3.42)	63	3.40 (2.94, 3.71)	39	0.0007
Birth Weight Centiles	47.4 (15.5, 81.5)	63	67.4 (38.0, 86.1)	39	0.092
Prematurity (<37 weeks gestation)	17 (27.0%)	63	2 (4.8%)	42	0.004
Small for Gestational Age (SGA)	10 (15.9%)	63	4 (10.3%)	39	0.42
Admission to NICU	11 (17.2%)	64	6 (14.3%)	42	0.69
Birth Complications	9 (14.1%)	64	7 (16.7%)	42	0.71
Gestational Age (weeks)	38 (36, 39)	63	40 (39, 40)	41	0.0001

The median gestational age at birth was significantly lower for CHEU (38 weeks) compared to CHUU (40 weeks; *p* < 0.0001), with a greater proportion of CHEU being born prematurely (27% vs. 4.8%, *p* = 0.004). While the median birthweight for CHEU (2.90 kg) was significantly lower than that of CHUU (3.40 kg; *p* = 0.0007), the median birthweight centiles did not differ significantly between groups (47.4 vs. 67.4 percentile; *p* = 0.092). A similar proportion of children in each group were classified as SGA at birth (CHEU: 15.9% vs. CHUU: 10.3%; *p* = 0.42). The proportion of children experiencing birth complications (CHEU: 14.1% vs. CHUU: 16.7%; *p* = 0.71) was similar between groups, as were the proportion of children admitted to NICU (CHEU: 17.2% vs. CHUU: 14.3%; *p* = 0.69). In the CHEU group, the majority of NICU admissions were due to preterm birth (54.5%, *n* = 6), with other reasons including maternal uterine diverticulum, heart murmur, breastfeeding issues, low glucose levels, and small size (each *n* = 1). In the CHUU group, reasons for NICU admission included meconium aspiration, jaundice, maternal diabetes (each *n* = 1), or other reasons (*n* = 3).

### 3.2 Univariate analyses of neurodevelopmental outcomes

The mean scores for each measure were in the average range for both the CHEU and CHUU (Table 2 and Supplementary Figures 1, 2). Within the CHEU group, a higher proportion of participants scored in the clinically impaired range (standardized score <70) on all measures (Table 2) than expected in the normative population, whereas 2% of individuals in the general population are expected to fall within these ranges. The proportion of CHEU falling in the clinically impaired range was significantly higher for the Working Memory Index (10.9% vs. 0.0%, *p* = 0.027) and was marginally higher for the Visual Spatial Index (7.7% vs. 0.0%, *p* = 0.07) as compared to CHUU.

**Table 2:** Neurodevelopmental outcomes in CHEU and CHUU: mean and confidence intervals of standard scores, and comparison of clinically impaired proportions.

Measure	CHEU mean (95%CI)	CHEU *n*	CHUU Mean (95%CI)	CHUU *n*	CHEU clinically impaired^1^ *n* (%)	CHUU clinically impaired^1^ *n* (%)	*p*-value^2^
Intellectual abilities							
Verbal comprehension index	95.78 (92.19, 99.37)	64	100.5 (96.2, 104.8)	42	2 (3.12%)	0 (0%)	0.25
Visual spatial index	90.12 (86.19, 94.06)	65	93.60 (89.63, 97.56)	42	5 (7.69%)	0 (0%)	0.07
Fluid reasoning index	94.38 (90.67, 98.10)	65	96.76 (91.63, 101.9)	42	5 (7.69%)	3 (7.40%)	0.92
Working memory index	90.86 (87.23, 94.49)	64	97.76 (93.57, 102.0)	42	7 (10.94%)	0 (0%)	0.027
Processing speed index	92.06 (88.04, 96.09)	63	95.19 (91.29, 99.09)	42	6 (9.52%)	1 (2.38%)	0.15
Full scale IQ	90.63 (86.96, 94.29)	64	96.10 (91.47, 100.7)	42	5 (7.81%)	3 (7.14%)	0.90
Language abilities							
Core language	92.69 (88.59, 96.79)	61	99.05 (94.75, 103.3)	41	6 (9.84%)	2 (4.88%)	0.36
Receptive language	90.70 (86.79, 94.62)	60	94.74 (89.87, 99.60)	38	4 (6.56%)	2 (5.26%)	0.79
Expressive language	94.43 (90.57, 98.30)	61	100.5 (96.15, 104.9)	38	2 (3.33%)	1 (2.63%)	0.84

^1^ Clinically impaired is defined as a score less than 70.

^2^ The *p*-value represents the comparison between the proportions of clinically impaired scores across groups.

Statistical comparisons by *χ*^2^ test.

CHEU, child who is HIV exposed uninfected; CHUU, child who is HIV unexposed uninfected; CI, confidence interval.

**Figure 1:**
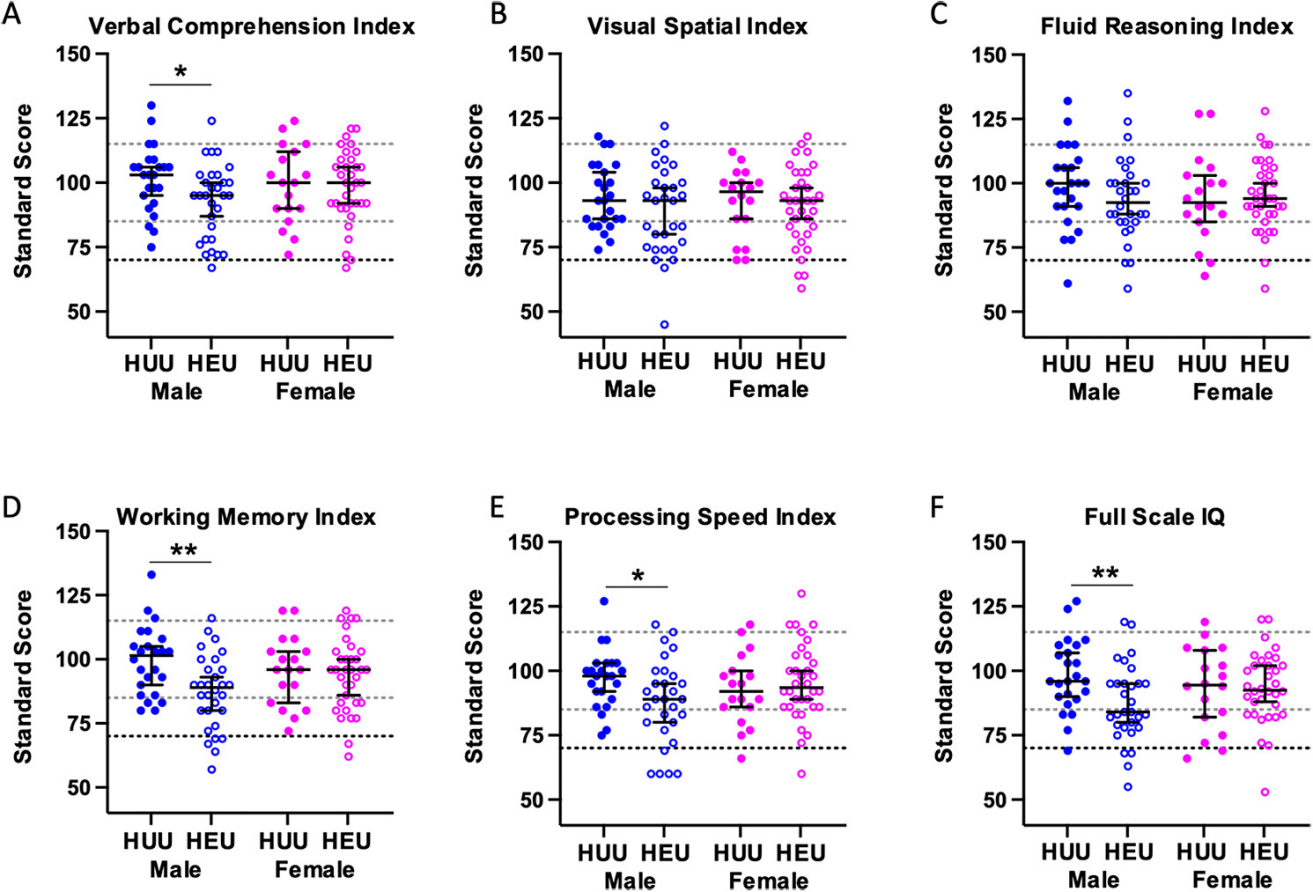
Standard scores of intellectual abilities for male and female CHEU compared to CHUU. Dot plot illustrating the distribution of standard scores for Verbal Comprehension **(A)** [CHEU (30 male, 34 female); CHUU (24 male, 18 female)], Visual-Spatial **(B)** [CHEU (30 male, 35 female); CHUU (24 male, 18 female)], Fluid Reasoning **(C)** [CHEU (30 male, 35 female); CHUU (24 male, 18 female)], Working Memory **(D)** [CHEU (30 male, 34 female); CHUU (24 male, 18 female)], Processing Speed **(E)** [CHEU (19 male, 34 female); CHUU (24 male, 18 female)], and Full-Scale IQ **(F)** [CHEU (30 male, 34 female); CHUU (24 male, 18 female)] indices in CHEU and CHUU, stratified by sex. Data are shown as dots for each individual participant with the mean and 95% confidence intervals indicated. Light dotted lines indicate ±1 standard deviation from the population mean of 100. The darker dotted line indicates 2 standard deviations below the population mean of 100. Dots falling below this line are classified as clinically impaired. Males are shown in blue, and females in pink. Closed circles indicate CHUU, and open circles indicate CHEU. *p < 0.05, **p < 0.01. Statistical comparisons by generalized linear models including an HEU-status*sex interaction term. CHEU, child who is HIV exposed uninfected; CHUU, child who is HIV unexposed uninfected. HUU, HIV unexposed uninfected; HEU, HIV exposed uninfected.

**Figure 2:**
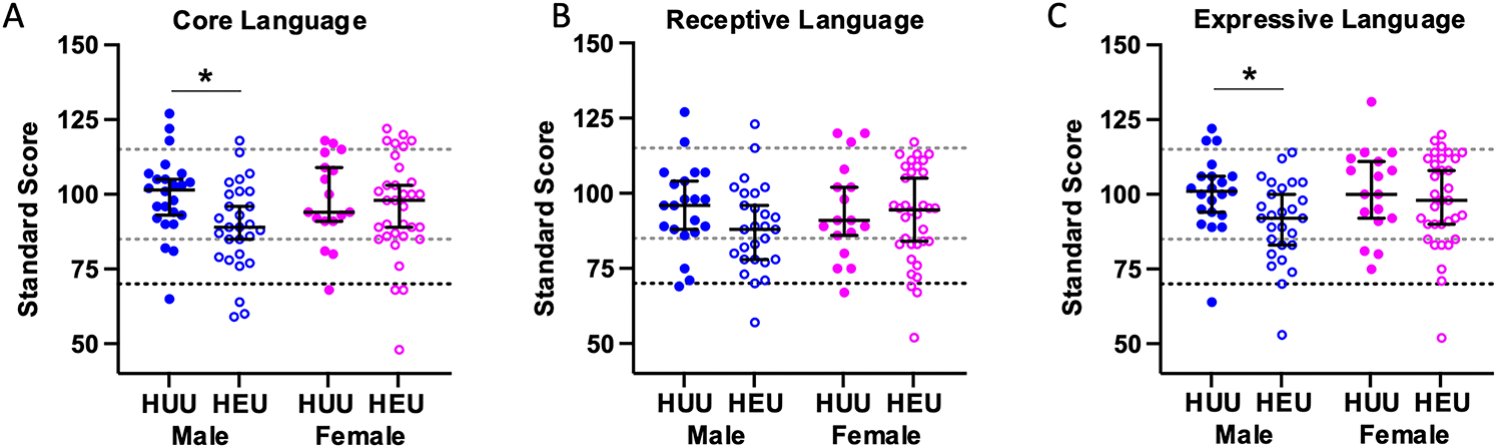
Standard scores of language abilities for male and female CHEU compared to CHUU. Dot plot illustrating the distribution of standard scores for Core Language **(A)** [CHEU (28 male, 33 female); CHUU (24 male, 17 female)], Receptive Language **(B)** [CHEU (27 male, 34 female); CHUU (21 male, 17 female)], and Expressive Language **(C)** [CHEU (27 male, 33 female); CHUU (21 male, 17 female)] indices in CHEU and CHUU, stratified by sex. Data are shown as dots for each individual participant with the mean and 95% confidence intervals indicated. Light dotted lines indicate ±1 standard deviation from the population mean of 100. The darker dotted line indicates 2 standard deviations below the population mean of 100. Dots falling below this line are classified as clinically impaired. Males are shown in blue, and females in pink. Closed circles indicate CHUU, and open circles indicate CHEU. *p < 0.05. Statistical comparisons by generalized linear models including an HEU-status*sex interaction term. CHEU, child who is HIV exposed uninfected; CHUU, child who is HIV unexposed uninfected. HUU, HIV unexposed uninfected; HEU, HIV exposed uninfected.

In the univariate analyses, CHEU had significantly lower mean standard scores for working memory (mean difference −6.90, *p* = 0.012), core language (mean difference −6.36, *p* = 0.030), and expressive language (mean difference −6.07, *p* = 0.035), and marginally lower for Full Scale IQ (mean difference −5.47, *p* = 0.06), compared to CHUU (Supplementary Table 4). Including sex as an interaction term in the models revealed that male CHEU were more vulnerable than female CHEU (Figures 1 and 2). Male CHEU had significantly lower mean standard scores for Verbal Comprehension (mean difference −8.72, *p* = 0.018), Working Memory (mean difference −12.12, *p* = 0.001), Processing Speed (mean difference −9.03, *p* = 0.017), Full Scale IQ (mean difference −10.83, *p* = 0.005), Core Language (mean difference −10.37, *p* = 0.007), and Expressive Language (mean difference −9.48, *p* = 0.011), and marginally lower for Receptive Language (mean difference −6.90, *p* = 0.092) (Figures 1 and 2, and Supplementary Table 5). Female CHEU had similar scores to female CHUU for all outcomes.

### 3.3 Multivariate analyses of neurodevelopmental outcomes- perinatal exposures and socioeconomic status

We next investigated how birth outcomes modified associations between HEU exposure status and the neurodevelopmental outcomes in multivariate models. Due to high correlation between perinatal variables (see methods) only gestational age at birth and being born SGA were considered. After controlling for these two variables, the differences in Verbal Comprehension were no longer significant in male CHEU. Working Memory (mean difference −12.14, *p* = 0.002) and Processing Speed indices (mean difference −9.17, *p* = 0.022), Full Scale IQ (mean difference −8.97, *p* = 0.027), and Core (mean difference −7.99, *p* = 0.041) and Expressive Language (mean difference −7.72, *p* = 0.048) scores remained significantly lower in male CHEU. Being SGA, regardless of HEU-status, was associated with significant declines in Processing Speed Index (mean difference −7.32, *p* = 0.047), Full Scale IQ (mean difference −9.03, *p* = 0.023), and Core (mean difference −9.31, *p* = 0.041) and Receptive (mean difference −9.97, *p* = 0.019) Language scores.

To account for socioeconomic status, we further controlled for household income in addition to gestational age at birth and SGA (Table 3). Household income was significantly associated with performance on all the cognitive measures except working memory. Including household income in the model led to a reduction in magnitude of all coefficients associated with HEU exposure status, with the exception of working memory (Table 3).

**Table 3:** Multivariate linear regression results for neurodevelopmental outcomes comparing groups, controlling for GA, SGA, and income.

Cognitive outcome	Predictor	Estimate	95% confidence interval	Predictor *p*-value
Verbal comprehension index	HEU status	−2.02	−8.49, 4.45	0.54
	Sex	2.92	−5.08, 10.92	0.47
	HEU × Sex (female)	2.97	−7.24, 13.18	0.57
	SGA	−1.77	−9.12, 5.57	0.64
	GA	0.68	−0.16, 1.53	0.11
	Income	4.65	2.91, 6.39	<0.0001
Visual spatial index	HEU status	−2.25	−10.20, 5.70	0.58
	Sex	1.31	−6.68, 9.31	0.75
	HEU × Sex (female)	1.25	−9.71, 12.20	0.82
	SGA	−3.90	−13.5, 5.71	0.43
	GA	−0.40	−1.41, 0.61	0.44
	Income	3.34	1.49, 5.20	<0.0001
Fluid reasoning index	HEU status	−1.20	−10.17, 7.77	0.78
	Sex	−0.59	−9.91, 8.72	0.90
	HEU × Sex (female)	3.14	−8.54, 14.83	0.60
	SGA	0.47	−11.09, 12.02	0.94
	GA	0.51	−0.46, 1.50	0.30
	Income	4.09	2.12, 6.05	<0.0001
Working memory index	HEU status	−10.80	−18.17, −3.44	0.004
	Sex	−1.13	−9.11, 6.84	0.78
	HEU × Sex (female)	8.34	−2.22, 18.91	0.12
	SGA	−3.70	−11.97, 4.56	0.38
	GA	0.21	−0.74, 1.18	0.65
	Income	1.83	−0.10, 3.77	0.06
Processing speed index	HEU status	−6.59	−14.62, 1.43	0.11
	Sex	−0.22	−7.69, 7.25	0.95
	HEU × Sex (female)	9.49	−1.16, 20.13	0.08
	SGA	−4.94	−12.38, 2.50	0.19
	GA	−0.03	−0.99, 0.93	0.95
	Income	3.31	1.07, 5.54	0.004
Full scale IQ	HEU status	−4.96	−12.29, 2.36	0.18
	Sex	1.05	−7.02, 9.12	0.80
	HEU × Sex (female)	6.15	−4.16, 16.46	0.24
	SGA	−4.80	−12.70, 3.11	0.23
	GA	0.44	−0.37, 1.24	0.20
	Income	4.93	3.07, 6.78	<0.0001
Core language	HEU status	−3.84	−11.06, 3.37	0.30
	Sex	4.83	−2.08, 11.74	0.17
	HEU × Sex (female)	1.19	−8.64, 11.01	0.81
	SGA	−5.35	−14.68, 3.98	0.21
	GA	0.76	0.05, 1.47	0.04
	Income	4.62	2.97, 6.27	<0.0001
Receptive language	HEU status	−0.95	−8.92, 7.01	0.81
	Sex	5.30	−3.02, 13.62	0.21
	HEU × Sex (female)	0.65	−9.97, 11.27	0.90
	SGA	−5.00	−13.07, 3.07	0.23
	GA	0.66	−0.18, 1.50	0.12
	Income	5.46	3.85, 7.06	<0.0001
Expressive language	HEU status	−5.27	−12.81, 2.27	0.17
	Sex	5.09	−2.60, 12.79	0.19
	HEU × Sex (female)	2.43	−8.09, 12.95	0.65
	SGA	−5.69	−15.82, 4.43	0.27
	GA	0.87	0.13, 1.61	0.02
	Income	3.41	1.64, 4.19	<0.0001

HEU, HIV exposed uninfected; SGA, small for gestational age; GA, gestational age; Income, household income.

*Note.* AIC = 8.00; BIC = 14624.48 for Verbal Comprehension model. AIC = 8.27; BIC = 19366.93 for Visual Spatial Model. AIC = 8.32; BIC = 20429.85 for Fluid Reasoning Model. AIC = 8.15; BIC = 17075.95 for Working Memory Model. AIC = 8.17; BIC = 17121.04 for Processing Speed Model. AIC = 8.03; BIC = 15103.00 for Full Scale IQ Model. AIC = 8.01; BIC = 14044.53 for Core Language Model. AIC = 8.06; BIC = 14388.67 for Receptive Language Model. AIC = 8.07; BIC = 14341.71 for Expressive Language Model.

## 4 Discussion

In this study, school-aged CHEU had lower mean scores on Working Memory and Expressive Language measures compared to CHUU. This is in contrast to previous studies that did not find these differences in infancy or early childhood (11, 17, 18, 21, 24, 25). Our results contribute to the understanding that cognitive deficits in CHEU may become more observable at later stages of childhood, as highlighted previously (23, 33–36). Importantly, our findings identify sex-specific vulnerabilities, with significant deficits being seen in male, but not female, CHEU in this cohort. Our results also highlight a distinct and persistent area of intrinsic challenge in working memory abilities, particularly for male CHEU, even after accounting for perinatal (gestational age at birth and SGA) and socioeconomic factors (income). Finally, our findings highlight the importance of economic factors, with household income being strongly associated with all neurodevelopmental measures, apart from Working Memory Index.

The sex-specific vulnerability observed in our study, even after controlling for perinatal risk factors, is a novel contribution to the literature. The few studies on school-aged CHEU (33, 35) have controlled for sex in their analysis rather than specifically examining for sex differences, and the limited research examining sex differences has not identified these discrepancies in younger groups (24, 25). Further, studies suggesting male vulnerabilities have not included comparisons with CHUU (37), hindering the generalizability of the findings, as without a comparison group, it can be speculated that males may be at a higher risk of neurodevelopmental deficits, regardless of HEU-status (38). These observed sex differences may indicate that male CHEU are more susceptible to maternal HIV and potentially ART exposures in the intrauterine environment. The intrauterine environment tends to be more pro-inflammatory for male fetuses compared to female fetuses (39, 40). Specifically, the male placenta has been shown to be less protective against inflammatory and infectious insults, which could make male fetuses more susceptible to *in utero* exposure to HIV and ARTs (39, 40). Studies have also shown that male and female fetuses respond differently to prenatal adversities, with male fetuses prioritizing physical growth at the expense of other organ processes. This may make male fetuses less adaptable to *in utero* changes, increasing the risk for adverse downstream effects, including on neurodevelopment (41). However, it is important to consider that societal and gender expectations may also contribute to the masking of more subtle language or cognitive challenges in girls that may not be captured through our standardized measures (42, 43).

Including perinatal factors (SGA and gestational age at birth) in the analyses led to only minor attenuations in HEU group differences. While factors such as prematurity and intrauterine growth restriction are known to influence early neurodevelopment, their long-term effects on cognition may be attenuated by other environmental factors as the child develops (10). In our study, household income had a substantial impact on intelligence and language outcomes in both CHEU and CHUU groups, highlighting the critical role of the broader socioeconomic environment in a child’s development over time. Socioeconomic status can impact neurodevelopment from conception through adulthood via direct and indirect pathways, such as perinatal exposure (e.g., nutrition), psychosocial stress, access to resources, educational opportunities, and cognitive stimulation (44). Further, the household and environmental context of CHEU is influenced by a series of indirect risks for child development, such as caregiver illness and hospitalization, and the mental health burden of HIV diagnosis (45, 46). These factors may limit social interaction and cognitive stimulation during early development, further impacting children’s cognitive, learning, and social development (47). The present finding raises the possibility of considering policies such as universal basic income, which is particularly relevant for the CHEU population where lower household income is more prevalent and income disparities are likely to compound neurodevelopmental challenges (23, 48). Further, the findings on household income can also indicate the impact of other related variables, such as single-parent households and additional socio-demographic challenges. Therefore, it is crucial to consider policies that address these broader socioeconomic disparities.

The most consistent finding of our study is that working memory deficits in CHEU persist even after controlling for perinatal risk factors and also socioeconomic status. In addition, the number of CHEU with clinically impaired Working Memory scores were significantly higher compared to CHUU, reflecting disparities between the groups (49). The observed working memory deficits align with the findings of a previous study on verbal working memory in school-aged CHEU (50). Working memory abilities may be more susceptible to the impacts of HIV and ART exposures in CHEU due to the prolonged development time of prefrontal lobes, and differences may become more evident in older children due to the later emergence of working memory and executive functioning abilities in childhood (49). Working memory is fundamental to learning and academic achievement (51), attentional control (52), development of cognitive abilities such as reasoning, problem-solving, decision-making (53), and social skills (54). Working memory deficits are also associated with broader executive functioning difficulties (55). These skills develop in later childhood and can be better assessed beginning at school age and into adolescence, when children have had more exposure to external factors, such as social and cognitive stimulation and learning environments. Working memory deficits may contribute to academic challenges observed in CHEU (56). Future research could further explore the association between working memory, executive functioning skills, and academic development in CHEU. Further, our findings suggest that a more extended follow-up period into middle childhood and adolescence is essential. Close follow-up of the CHEU population in the school-age period provides an opportunity for early intervention to improve broader executive functioning during these sensitive developmental stages.

Several limitations must be acknowledged. Our CHEU and CHUU samples are relatively small, impacting the statistical power to conduct between-sex analyses and the generalizability of the findings. In addition, recruitment bias might have impacted our findings, as parents suspecting neurodevelopmental challenges may have been more inclined for their children to participate in the study; however, this is unlikely to explain the observed sex differences. Further, while our study revealed a higher risk of working memory deficits in CHEU, we were unable to provide a precise mechanism underlying this association. Previous literature has identified associations between specific ART regimens and language outcomes (19), however, due to sample size limitations, we did not explore these associations. Finally, we are only able to present findings from assessment at a single time point. Tracking neurodevelopmental trajectories longitudinally would be more informative and is planned as part of the larger KIND study.

In conclusion, our study contributes to the growing body of research on the neurodevelopmental outcomes of CHEU. We found neurodevelopmental vulnerabilities in school-aged CHEU, with male CHEU showing greater vulnerability for poorer intellectual and language outcomes. Household income was identified as a contributing factor for most of these neurodevelopmental challenges, but even after controlling for income, working memory deficits persisted in CHEU, highlighting a poorer outcome due to HEU status. Our findings emphasize the need for close neuropsychological follow-up in this population beyond five years of age and potential early interventions and supports, particularly for CHEU males and those born into low-income households. Further, our findings highlight the necessity for future research to continue to explore the complex interplay between biological and psychosocial factors, HIV and ART exposure, brain development, and neurodevelopmental outcomes in CHEU.

## Data Availability

The raw data supporting the conclusions of this article will be made available by the corresponding author upon reasonable request.

## KIND study team

Dr. Ari Bitnun, Dr. Jason Brophy, Jennifer Bowes, Dr. Shreya Dhume, Dr. Eve Forster, Dr. Marieve Hurtubise, Haoua Inoua, Majorie Kabahenda, Cassandra Kapoor, Dr. Elka Miller, Dr. Stephanie León, Dr. Jason Lerch, Dr. Lena Serghides, Dr. John Sled, Dr. Mary Lou Smith, Nicci Stein, Dr. Michael Szego, Dr. Margot Taylor, Dr. Julia Young, Dr. Mark Yudin.

## KIND study staff

Cheryl Arneson, Jennifer Brazeau, Meaghan Hall, Matt Head, Victoria Kim, Dr. Tamara Tavares, Bilal Syed, Lindsey Ure, Tharaniya Kumarathasan.
