# Supplementary material for: Intelligence and language outcomes in school-aged children who are HIV-exposed, uninfected: the role of sex, perinatal risk factors, and socioeconomic status
Source: Front Pediatr. 2025 Jul 17;13:1540420. doi: 10.3389/fped.2025.1540420 (PMC12310716; doi:10.3389/fped.2025.1540420)
Supplement: Supplementary file 1 [file Datasheet1.pdf]

Supplementary Material

1 Supplementary Tables

Supplementary Table 1. Correlations between intellectual and language outcomes, and birth weight and gestational age, with the perinatal and socio-economic factors

	GA	Birth Weight Centiles	BW	SGA	Prematurity	Admission to NICU	Birth Complications	Household Income	Maternal Education
**Cognitive Outcome**									
**Intellectual Abilities**									
Verbal Comprehension Index	0.16	0.17	0.23	-0.22	-0.24	0.02	0.13	0.45	0.29
Visual Spatial Index	-0.04	0.02	-0.01	-0.27	0.05	0.22	0.19	0.32	0.23
Fluid Reasoning Index	0.11	-0.01	0.10	-0.10	-0.16	0.21	0.02	0.38	0.36
Working Memory Index	0.11	0.18	0.25	-0.24	-0.12	0.04	0.20	0.25	0.24
Processing Speed Index	0.03	0.17	0.16	-0.28	-0.08	0.10	0.34	0.33	0.24
Full Scale IQ	0.12	0.16	0.22	-0.34	-0.16	0.08	0.19	0.49	0.39
**Language Abilities**									
Core Language	0.20	0.22	0.31	-0.30	-0.21	0.05	0.14	0.46	0.36
Receptive Language	0.14	0.13	0.22	-0.25	-0.31	-0.11	0.18	0.39	0.41
Expressive Language	0.20	0.18	0.27	-0.29	-0.16	0.20	0.04	0.36	0.28
**Perinatal Factors**									
Gestational Age (GA)	1.00	0.11	0.69	0.14	-1.00	-0.66	-0.75	0.06	-0.08
Birth Weight (BW)	0.69	0.75	1.00	-0.58	-0.85	-0.69	-0.29	0.11	0.11

Pearson's r for continuous variables, the Phi coefficient for binary variables, and point-biserial correlation for binary-continuous variable pairs are shown.

Supplementary Table 2. Generalized linear model results for neurodevelopmental outcomes by HEU status with sex interaction term adjusting for gestational age at birth

Cognitive Outcome	Predictor	Estimate	Confidence Interval	p-value
**Intellectual Abilities**				
Verbal Comprehension Index	HEU status	-6.81	-14.22, 0.60	0.072
	Sex	-2.30	-10.82, 6.22	0.60
	HEU x Sex (female)	6.92	-4.14, 17.99	0.22
	GA	0.66	-0.39, 1.71	0.22
Visual Spatial Index	HEU status	-5.72	-14.29, 2.84	0.19
	Sex	-1.81	-9.69, 6.08	0.65
	HEU x Sex (female)	2.65	-8.52, 13.81	0.64
	GA	-0.50	-1.69, 0.69	0.41
Fluid Reasoning Index	HEU status	-4.93	-14.37, 4.52	0.31
	Sex	-5.51	-15.56, 4.54	0.28
	HEU x Sex (female)	6.37	-6.14, 18.89	0.32
	GA	0.51	-0.63, 1.64	0.38
Working Memory Index	HEU status	-12.95	-20.64, -5.27	**0.001**
	Sex	-4.28	-12.50, 3.93	0.31
	HEU x Sex (female)	11.32	0.51, 22.13	**0.04**
	GA	0.096	-0.89, 1.08	0.85
Processing Speed Index	HEU status	-9.86	-17.77, -1.94	**0.015**
	Sex	-4.72	-12.42, 2.98	0.23
	HEU x Sex (female)	12.11	1.04, 23.18	**0.032**
	GA	-0.10	-1.22, 1.03	0.867
Full Scale IQ	HEU status	-10.36	-18.64, -2.09	**0.014**
	Sex	-5.15	-14.31, 4.01	0.27
	HEU x Sex (female)	10.74	-0.92, 22.42	0.071
	GA	0.33	-0.76, 1.41	0.55
**Language Abilities**				
Core Language	HEU status	-8.77	-16.77, -0.76	**0.032**
	Sex	-1.54	-10.16, 7.07	0.72
	HEU x Sex (female)	7.03	-4.66, 18.72	0.24
	GA	0.76	-0.19, 1.70	0.12
Receptive Language	HEU status	-5.87	-14.77, 3.03	0.20
	Sex	-1.49	-11.18, 8.2	0.76
	HEU x Sex (female)	4.98	-7.41, 17.36	0.43
	GA	0.50	-0.56, 1.56	0.35
Expressive Language	HEU status	-8.33	-16.31, -0.34	**0.041**
	Sex	0.24	-8.41, 8.89	0.96
	HEU x Sex (female)	5.87	-5.51, 17.26	0.31
	GA	0.73	-0.16, 1.62	0.11

Supplementary Table 3. Generalized linear model results for neurodevelopmental outcomes by HEU status with sex interaction term adjusting for being born SGA and gestational age at birth

Cognitive Outcome	Predictor	Estimate	Confidence Interval	p-value
**Intellectual Abilities**				
Verbal Comprehension Index	HEU status	-5.93	-13.25, 1.38	0.11
	Sex	-0.89	-9.52, 7.75	0.84
	HEU x Sex (female)	5.38	-5.71, 16.48	0.34
	SGA	-5.76	-12.90, 1.37	0.11
	GA	0.77	-0.27, 1.81	0.15
Visual Spatial Index	HEU status	-4.70	-12.91, 3.51	0.26
	Sex	-1.42	-9.68, 6.84	0.74
	HEU x Sex (female)	2.09	-9.14, 13.32	0.72
	SGA	-6.75	-15.98, 2.48	0.15
	GA	-0.39	-1.54, 0.76	0.50
Fluid Reasoning Index	HEU status	-4.53	-13.86, 4.79	0.34
	Sex	-3.95	-13.97, 6.08	0.44
	HEU x Sex (female)	4.73	-7.65, 17.12	0.45
	SGA	-2.92	-14.40, 8.56	0.62
	GA	0.59	-0.50, 1.68	0.29
Working Memory Index	HEU status	-12.14	-19.68, -4.59	**0.002**
	Sex	-2.62	-10.76, 5.51	0.53
	HEU x Sex (female)	9.54	-1.15, 20.23	0.080
	SGA	-5.57	-13.78, 2.64	0.18
	GA	0.22	-0.75, 1.19	0.66
Processing Speed Index	HEU status	-9.17	-17.04, -1.30	**0.022**
	Sex	-2.95	-10.38, 4.49	0.44
	HEU x Sex (female)	10.38	-0.42, 21.18	0.060
	SGA	-7.32	-14.53, -0.11	**0.047**
	GA	0.053	-1.05, 1.15	0.92
Full Scale IQ	HEU status	-8.97	-16.92, -1.01	**0.027**
	Sex	-2.98	-11.90, 5.94	0.51
	HEU x Sex (female)	8.39	-2.95, 19.72	0.15
	SGA	-9.03	-16.80, -1.27	**0.023**
	GA	0.51	-0.55, 1.56	0.35
**Language Abilities**				
Core Language	HEU status	-7.99	-15.65, -0.34	**0.041**
	Sex	0.96	-7.19, 9.11	0.82
	HEU x Sex (female)	4.92	-6.23, 16.07	0.39
	SGA	-9.31	-18.23, -0.40	**0.041**
	GA	0.90	-0.02, 1.82	0.055
Receptive Language	HEU status	-5.03	-13.77, 3.70	0.26
	Sex	0.82	-8.76, 10.40	0.87
	HEU x Sex (female)	3.04	-9.05, 15.12	0.62
	SGA	-9.97	-18.31, -1.63	**0.019**
	GA	0.66	-0.36, 1.69	0.21

Expressive Language	HEU status	-7.72	-15.38, -0.06	**0.048**
	Sex	2.29	-6.10, 10.68	0.59
	HEU x Sex (female)	4.17	-6.86, 15.20	0.46
	SGA	-8.69	-18.35, 0.97	0.078

Supplementary Table 4. Generalized linear model results for neurodevelopmental outcomes by HEU status

Cognitive Outcome	Predictor	Estimate	Confidence Interval	p-value
**Intellectual Abilities**				
Verbal Comprehension Index	HEU status	-4.72	-10.15, 0.71	0.089
Visual Spatial Index	HEU status	-3.47	-8.90, 1.95	0.21
Fluid Reasoning Index	HEU status	-2.38	-8.51, 3.76	0.45
Working Memory Index	HEU status	-6.90	-12.28, -1.53	**0.012**
Processing Speed Index	HEU status	-3.13	-8.57, 2.31	0.26
Full Scale IQ	HEU status	-5.47	-11.19, 0.25	0.06
**Language Abilities**				
Core Language	HEU status	-6.36	-12.12, -0.60	**0.030**
Receptive Language	HEU status	-4.03	-10.06, 2.00	0.19
Expressive Language	HEU status	-6.07	-11.69, -0.44	**0.035**

Supplementary Table 5. Generalized linear model results for neurodevelopmental outcomes by HEU status with sex interaction term

Cognitive Outcome	Predictor	Estimate	Confidence Interval	p-value
**Intellectual Abilities**				
Verbal Comprehension Index	HEU status	-8.72	-15.93, -1.50	**0.018**
	Sex	-2.53	-11.04, 5.99	0.56
	HEU x Sex (female)	8.01	-2.95, 18.98	0.15
Visual Spatial Index	HEU status	-5.14	-12.98, 2.70	0.20
	Sex	-1.82	-9.56, 5.92	0.64
	HEU x Sex (female)	3.47	-7.51, 14.45	0.53
Fluid Reasoning Index	HEU status	-5.17	-13.63, 3.30	0.23
	Sex	-4.83	-14.81, 5.14	0.34
	HEU x Sex (female)	6.17	-6.24, 18.57	0.33
Working Memory Index	HEU status	-12.12	-19.43, -4.79	**0.001**
	Sex	-3.86	-11.95, 4.22	0.35
	HEU x Sex (female)	10.56	-0.09, 21.21	0.052
Processing Speed Index	HEU status	-9.03	-16.45, -1.60	**0.017**
	Sex	-4.12	-11.81, 3.56	0.29
	HEU x Sex (female)	11.78	0.89, 22.68	**0.034**
Full Scale IQ	HEU status	-10.83	-18.48, -3.19	**0.005**
	Sex	-4.83	-13.88, 4.21	0.30
	HEU x Sex (female)	11.03	-0.44, 22.50	0.059
**Language Abilities**				
Core Language	HEU status	-10.37	-17.90, -2.85	**0.007**
	Sex	-1.39	-9.86, 7.08	0.75
	HEU x Sex (female)	7.75	-3.76, 19.26	0.19
Receptive Language	HEU status	-6.90	-14.93, 1.13	0.092
	Sex	-1.44	-10.92, 8.05	0.77
	HEU x Sex (female)	5.43	-6.69, 17.55	0.38
Expressive Language	HEU status	-9.48	-16.79, -2.17	**0.011**
	Sex	0.37	-8.15, 8.89	0.93
	HEU x Sex (female)	6.13	-5.09, 17.37	0.28

2 Supplementary Figures

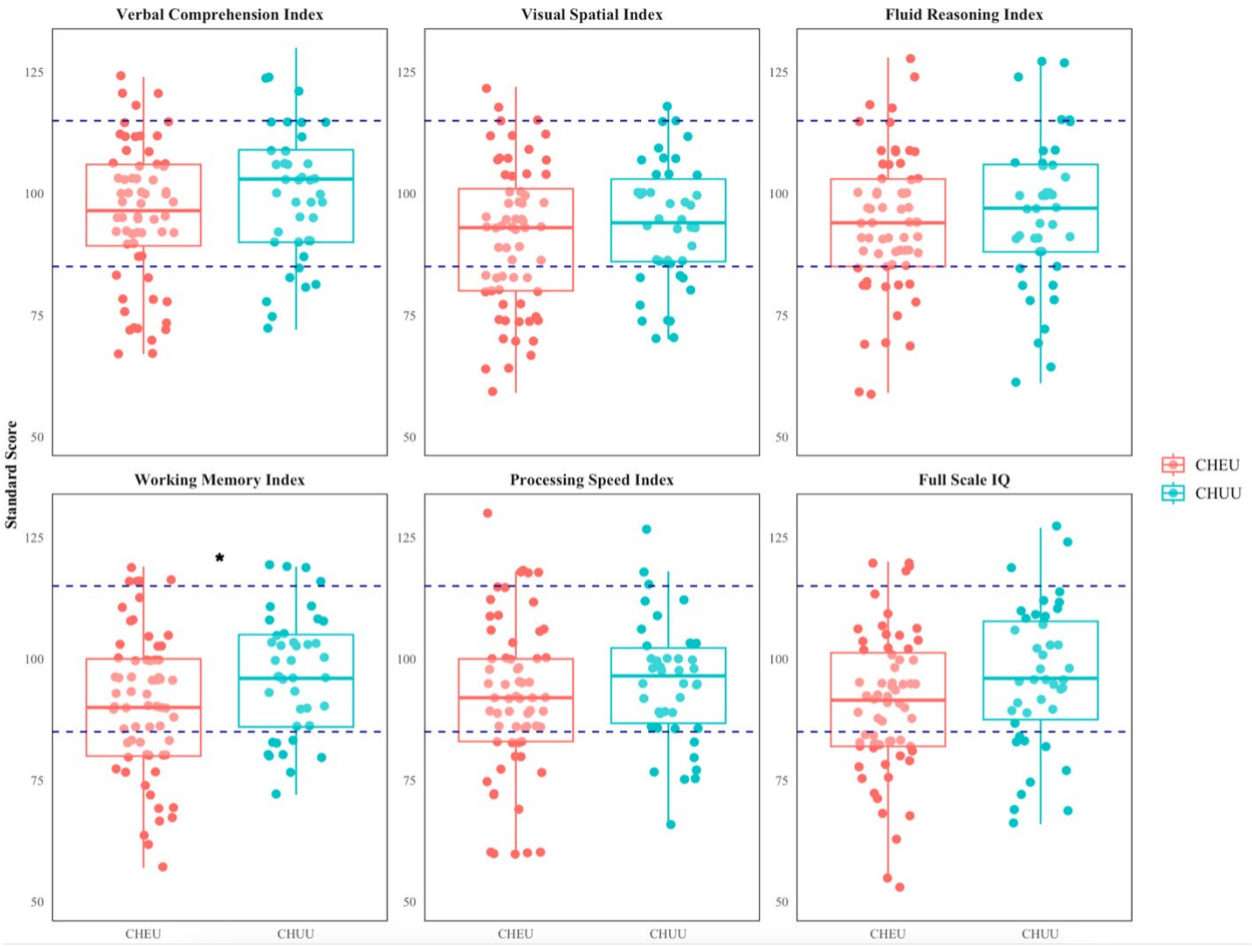

Supplementary Figure 2. Box plot illustrating the distribution of the standard scores of Verbal Comprehension, Visual-Spatial, Fluid Reasoning, Working Memory, Processing Speed, and Full-Scale IQ indices in CHEU and CHUU. The data points represent individual scores for each child within both groups. The box represents the medians and IQRs. The horizontal lines represent the range of standard scores within 1 SD of the mean (85–115). * $p<0.05$. CHEU demonstrated statistically significantly ($p=0.012$) lower mean scores than CHUU on the measure of Working Memory.

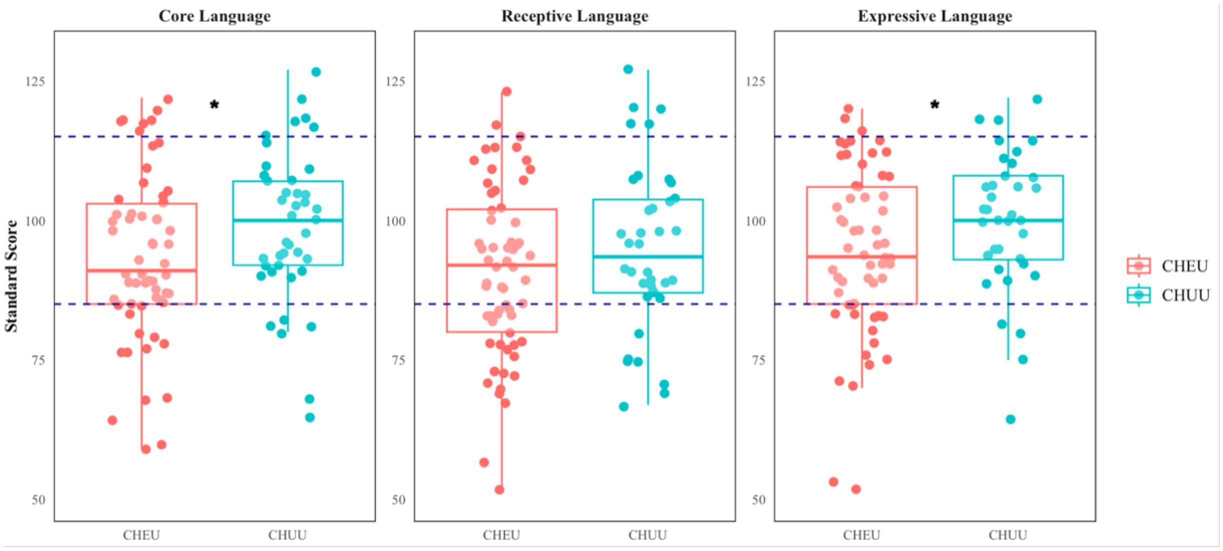

Supplementary Figure 2. Box plot illustrating the distribution of the standard scores of Core Language, Receptive Language, and Expressive Language indices in CHEU and CHUU. The data points represent individual scores for each child within both groups. The box represents the medians and IQRs. The horizontal lines represent the range of standard scores within 1 SD of the mean (85–115). * $p<0.05$. CHEU demonstrated statistically significantly lower mean scores than CHUU on the measure of Core Language ($p=0.030$) and Expressive Language ($p=0.035$).
